# Students’ perception of educational environment based on Dundee Ready Education Environment Measure and the role of peer mentoring: a cross-sectional study

**DOI:** 10.1186/s12909-022-03219-8

**Published:** 2022-03-15

**Authors:** Shadab Behkam, Amirhossein Tavallaei, Nastaran Maghbouli, Mahboobeh Khabaz Mafinejad, Jemal Haidar Ali

**Affiliations:** 1 Mentoring Office, Educational Deputy, School of Medicine, Tehran University of Medical Sciences, Tehran, Iran; 2 Department of Physical Medicine and Rehabilitation, School of Medicine, Tehran University of Medical Sciences, Tehran, Iran; 3 Department of Medical Education, Education Development Center (EDC), Health Professions Education Research Center, Tehran University of Medical Sciences, Tehran, Iran; 4 Addis Ababa University,College of Health Sciences, School of Public Health Addis Ababa University, Addis Ababa, Ethiopia

**Keywords:** Curriculum, Mentoring, Medical students, Reform, Iran

## Abstract

**Objective:**

The curricular reform at Tehran University of Medical Sciences (TUMS), Iran, has been implemented since 2011 when peer mentoring program started. The program is believed to have a crucial role in students’ perception of the educational environment (EE). We aimed to determine how students perceive the educational environment and compared the mentees and non-mentees’ perception of EE.

**Methods:**

A cross-sectional descriptive study was conducted among 190 first-year medical students enrolling at Tehran University of Medical Sciences from March to September 2019. A questionnaire was used to collect information on students’ age, gender, marital status, dormitory status, and their mentoring status including satisfaction of mentor-mentee relationship. The study also employed Dundee Ready Education Environment Measure (DREEM). The collected data were then entered and analysed using SPSS version 20. To compare the perception of EE between mentees and non-mentees, we used independent t-test.

**Results:**

The mean (SD) for total DREEM score for EE was 144.1 (19.3), which signifies a more positive than negative educational environment perception. Nonetheless, the mean scores of total DREEM was not significantly different between students with or without mentors (*P *=0.390). The overall mean score for student perceptions of learning for mentees was 32.47 (4.5) while for those without a mentor, the score was 31.70 (4.9) (*P *=0.491). The items concerned with “emphasizing factual learning” and “teacher-based teaching” were rated the least. The item “having an appropriate support system” was scored significantly different between students with or without mentors (*P *=0.009).

**Conclusions:**

Since having an appropriate support system was significantly different between groups, we suggest curriculum designers focus on the above-mentioned issue under caption for improvement during the reform programs.

**Supplementary Information:**

The online version contains supplementary material available at 10.1186/s12909-022-03219-8.

## Background

Educational environment (EE) is a complex concept consisting of diverse physical places, contexts, and cultures in which students experience learning activities [1]. Worldwide, medical educators are trying to recognize EE’s components and their interactions to reform EEs for added productivity [2]. In addition to curriculum and technical support, other factors are evidently of great significance in medical schools environment, including student services, mentor support, communication skills development, and self-efficacy [3]. A growing number of studies refer to mentoring programs as part of the curricular reform or its supportive mechanism and ensure that such changes are not likely to harm students’ learning experiences [4–6].

The Federation for Medical Education (WFME) believes that educational environment affects students’ knowledge, the motivation to learn, personal safety, and their well-being. EE is an important indicator for the evaluation and change of medical education programs [2]. The proposed EE instruments like CUES (College and University Environment Scales) [7], CLES (Classroom Environment Scales) [8], and CUCEI (College and University Environment Inventory) [9] have been used for learning environment evaluation for decades. However, they have not been used for medical education due to differences in goals and strategies. All the same, DREEM scale was developed [10] and has ever since been used widely across various academic formal settings in medical education. However, the heterogeneity in reporting and analysis of this tool found in different studies has made the comparison between programs very challenging [11]. Receiving a comprehensive systemic feedback both from faculty members and from students experiencing educational process is essential because the student’s self-perception is assumed to have impressive impact on the expected probability of success in reform [12]. Evidence from a qualitative study shows that cooperation with administration and professors, acceptance by the social community, sources of support, and competitive environment are amongst the key components of EE to be considered in this regard [12, 13]. It is worth noting that, in the literature and in certain studies, educational environment and learning environment are terms which are often used interchangeably [14–16]. In this study, we used the term educational environment to evaluate perceptions of learning, perceptions of teachers, academic self-perception, perceptions of atmosphere, and social self-perceptions [17]. Like some previous studies, we have also used DREEM which employs both terms, i.e. learning environment [18, 19] and educational environment [20, 21] .

Previous studies consider peer-mentoring programs as a potentially effective factor in different domains of the educational environment such as students learning efficacy [22], student-professor communication [23] and students’ social and academic self-perception [24]. In their study conducted in four European countries, Antohe et al. explored the situation of clinical placements for student nurses and assessed students’ satisfaction with the learning environment. They documented that their greater satisfaction with their clinical placements strongly correlated with the supervisory model. Interestingly, the most satisfied students were those who had the experience of individualised supervision. This finding signifies that the individualized supervision model is a crucial factor in students’ total satisfaction during their clinical training periods [25]. Another study documented also that peer-mentoring program and academic atmosphere as perceived by the students were correlated significantly. In fact, the students with peer-mentors had positive ideas towards their achievement and appreciated the educational goals of the institution more [26].

A range of factors like students’ academic level, achievements [27], gender [28], quality of life, resilience [29], positive attitudes towards course, mindfulness [30], preparation for practice and psychological distress [31], were assessed in relationship with educational environment in different studies. Nonetheless, as yet, there have been no studies about the relationship between medical students’ perception about educational environment and the role of peer mentoring. Furthermore, pragmatically, participating in peer mentoring programs seems to have a crucial influence on the student perception and satisfaction [32, 33]. However, the program requires detailed feedback from medical students regarding their mentoring experiences, especially the effect on educational setting. The aim is to provide a beneficial guide for strategic planning and to learn about corrective actions in items that students had negative perceptions about them.

With this background information, we aimed to examine two research questions: (a) Is the perception of EE different in students with and without peer-mentors? and (b) Do the subscales or items of EE differ between these two groups? The aim is to find out how students perceive the educational environment and to compare the mentees’ and non-mentees’ perceptions of EE.

## Methods

### Study setting

A cross-sectional descriptive-analytical study was carried out in the faculty of medicine, Tehran University of Medical Sciences between March and September 2019 to assess the learning environment and educational atmosphere using DREEM. The traditional curriculum of medical faculty consists of three phases: the first phase (5 semesters) for basic sciences; the second phase (2 semesters) for diseases pathophysiology courses; and the third phase (8 semesters) for a clinical internship using lecture-based classes and bed-side education with teacher having the key role. Since 2011, the first two phases changed in order to place more emphasis on early clinical exposure. The integration proved to be beneficial not only for basic science content but also for clinical theory classes, transferring critical thinking and problem-solving skills through case-based classes, problem-based learning (PBL), team-based learning (TBL), and workshops in combination with a conventional system in some cases. Considering curriculum changes, administrants took measures to encourage active participation in clinical settings, to provide opportunities for supervised tasks and lectures, improving behavioral and psychosocial topics of the existing curriculum and employing more interactive teaching methods [34]. We received some feedbacks on shortcomings of mentioned sections through mentee-mentors interactions. Therefore, in addition to examining peer mentoring role, we decided to design this study to evaluate “real” perception of students after 7 years of new curriculum implementation.

### Participants

A total of 190 first-year medical students enrolled for present study. To calculate the sample size, we used the G*Power 3.1.9.7 software [35, 36]. A sample of 152 students was adequate assuming α = 0.05, β = 0.2, population proportion:50% and population size of 250.

### Procedure

Along with the above-mentioned reforms, the mentoring program was meant to assist the first-year medical students to adjust with the school environment at TUMS from 2011 to 2019. One hundred and eighty-three mentors (3rd and 4th -year medical students) were educated to guide the junior students through 8 years of mentoring program implementation. To qualify for mentorship, students applied voluntarily. The administrants made sure that their academic performance was good enough and they were also in good health/professional conditions. Then their applications were evaluated based on their previous history of teaching roles and a self-assessment of mentorship skills. Following this process, senior mentors were assigned to the selected applicants based on their merits as judged by the members of the faculty. After the selection process was completed, all mentors were trained on the concept of mentoring, communication skills, program rules, and expectations through a three-day workshop. After a brief description of the program, the medical students as mentees registered for the program voluntarily by filling out the forms. They were assigned to mentors based on gender, ethnicity, living place, and scientific background. Mentees received mentorship through one-on-one meetings, predominantly face to face. About 1002 first-year medical students participated as mentees through 8 years. Two hundred and fifty first-year entrants, regardless of being mentees or not, requested to participate in this study during academic year 2018/2019.

### Data collection

Baseline demographic data including age, gender, marital status, dormitory status, and mentoring-status-related data (showing if they had a mentor or not and if they were satisfied with the mentor-mentee relationship based on 5-point likert scale items) were collected from all participants who were present at class and agreed to fill the forms. To increase the response rate, we handed out forms personally to the students and briefed them on the purpose of the study and consequently all filled the forms voluntarily. A short message was sent as a reminder 3 days later. Filling out questionnaires took 10 to 15 minutes and forms that had incomplete information were discarded.

### Measurement tools

One of the obstacles was lack of consensus regarding an obvious and operational description of educational environment concept [37, 38]. Therefore, it was crucial to use a wide-ranging, valid and reliable questionnaire. One of the most widely used questionnaires for this purpose is the Dundee Ready Education Environment Measure (DREEM) which has proved to be the best tool for evaluating medical students’ perceptions of the learning environment [14]. DREEM instrument was used to measure the educational learning environment from the perspective of students. It consisted of 50 closed question statements and five domains including perceptions of learning, perceptions of teachers, academic self-perceptions, perceptions of atmosphere, and social self-perceptions [11]. Twelve items (max score: 48) evaluated students’ perception of learning (SPL), eleven (max score: 44) measured their perceptions of teachers (SPT), eight (max score: 32) estimated their academic self-perceptions (SASP), twelve items (max score: 48) were used to determine the students’ perceptions of atmosphere (SPA), and, finally, seven (max score: 28) aimed to assess their social self-perceptions (SSSP). The DREEM items rated from 4 to 0, namely “Strongly agree” (4), “Agree” (3), “Unsure” (2), “Disagree” (1), and “Strongly disagree” (0). Reverse scoring was required for items 4, 8, 9, 17, 25, 35, 39, 48, and 50. According to the Association for Medical Education in Europe (AMEE) interpreting guide [39], the maximum score is 200 points and a score of 51-100 indicates ‘‘plenty of problems” while a score of 101 to 150 is ‘‘more positive than negative’’ and a score of 100 is interpreted as ‘‘considerable ambivalence by students and needs to be improved.’’ Items with mean scores of less than two should be closely considered. Except for negative items mentioned, a higher score means better interpretation. The validity and reliability of the Persian translation of DREEM were proved and was found to be excellent with a Cronbach’s alpha coefficient of 0.91 by Jalili et al [40].

### Statistical analysis

The collected data were entered and analysed using SPSS statistical package program version 20 (SPSS Inc., Chicago, IL, USA). The internal consistency reliability of the scale was determined using Cronbach’s alpha and was 0.713 for the total subscale (Table 1).

**Table 1:** Internal consistency reliability of DREEM scale and subscales

Subscale	Number of items	Cronbach’s Alpha
SPL	12	0.703
SPT	11	0.741
SASP	8	0.753
SPA	12	0.568
SSSP	7	0.697
Total DREEM	50	0.713

The detail is shown in the response form. Means and standard deviations, total scores, and subscale scores were calculated. Although a categorical variable with five levels such as Likert items cannot be normally distributed, one could argue that the sums of independent items, including scales, are likely to be less skewed and more normally distributed than the items themselves. Treating the data from scales as ordinal prevents the use of more potent modes of analysis [41]. The Shapiro-Wilk test was used to determine whether the DREEM total score was distributed normally or not. Although normality can be explored numerically, visually, and statistically, in this study, DREEM total scores are distributed normally statistically (*P *=0.358). Consequently an Independent T-test and ANOVA were used to compare the means of quantitative variables [41]. Nonetheless, large datasets may result in significant tests of normality when the distribution graphs look fairly normal while small datasets reduce the statistical power to detect non-normality and therefore they require careful interpretation. Overall, *P*-value of less than 0.05 was considered statistically significant except for DREEM items. Mean differences between two groups where P was less than 0.01 (*P *<0.01) was considered statistically significant for each DREEM items.

## Results

### Demographic statistics

Out of 190 questionnaires distributed in the study, 169 students completed the questionnaires with a response rate of 89.0%. Incomplete forms were excluded from the study and this made the item response rate (IRR) 100%. Table 2 depicts the demographic and educational characteristics of participants. The mean age of participants was 20.2 (SD 1.4). Eighty-seven (51.5%) medical students were as mentees in this study. Our results showed that the total DREEM mean score was not different based on gender (*P* = 0.188), dormitory status (*P* = 0.268), marital status (*P* =0.243), and age (*P* = 0.365).

**Table 2:** Demographic and educational characteristics of participants based on being a mentee

	Frequency (percentage) of participating students	Frequency (percentage) of students as mentees	*p*-value of difference in mentor possession rate per characteristic
**Age ( in mean(SD))**	20.2 (1.4)	20.1(1.5)	0.365
**Gender**			0.188
Male	86 (50.9%)	40 (46.0%)	
Female	83 (49.1%)	47 (54.0%)	
**Dormitory status**			0.268
living in dormitory	71 (42.0%)	33 (38.0%)	
living with family	98 (58.0%)	54 (62.0%)	
**Marital status**			0.243
Married	2 (1.2%)	0 (0.0%)	
Single	167 (98.8%)	87 (100.0%)	

### Overall subgroup analysis

In Table 3, nine items that earned a mean score of less than two and items with score > 4 are shown as weaknesses and strengths of TUMS-EE. The mean (SD) for total DREEM score was 144.1 ( 19.3) which signifies a more positive than negative educational environment perception. The mean scores of total DREEM between the groups that had mentors and students without mentors was not statistically significant (*P *=0.390).

Our results showed that total DREEM mean score was not different based on gender (*P* = 0.177), dormitory status (*P* = 0.380), marital status (*P* =0.819), and age (*P* = 0.432).

**Table 3:** Items with mean score <2 and >4 as program weaknesses and strengths

Item	Subscale	Mean score
Mean score < 2		
48. The teaching is too teacher-centered	SPL	0.7
25. The teaching over-emphasizes factual learning	SPL	0.4
9. The teachers are authoritarian	SPT	1.7
8. The teachers ridicule the students	SPT	1.6
39. The teachers get angry in class	SPT	0.9
50. The students irritate the teachers	SPA	1.4
35. I find the experience disappointing	SPA	1.3
17. Cheating is a problem in this school	SPA	1.5
4. I am too tired to enjoy the course	SSSP	1.1
Mean score > 4		
2. The teachers are knowledgeable	SPT	4.1
15. I have good friends in this school	SSSP	4.1

*SPL* Student Perception of Learning, *SPT* Student Perception of Teachers, *SASP* Students’ academic self-perceptions, *SPA* Students’ perceptions of atmosphere, *SSSP* Students’ social self-perceptions

#### Perceptions of learning

Mean scores and statistically significant differences of the DREEM subscales and items based on having a mentor are shown in Tables 4 and supplementary Table 1. The mean (SD) of perceptions of Learning for students with a mentor was 32.47/48 while for those without a mentor, the score was 31.70/48 (*P *=0.491). Items “The teaching over-emphasizes factual learning” and “The teaching is too teacher-centered” earned mean score less than 2. The mean scores of items “The teaching is sufficiently concerned to develop my competence”, “The teaching is well focused”,” I feel I am being well prepared for my profession”,” The teaching time is put to good use” and “I am clear about the learning objectives of the course” were more than 3. No item was different between two groups considering *p *<0.001 for statistical significance of mean differences.

**Table 4:** Total DREEM and subscales score differences between groups of students with and without a mentor

DREEM Subscales	DREEM mean scores of students mean (SD)		*P*-value	Cohen’s d
	with mentor	without mentor		
SPL	32.4 (6.5)	31.7 (6.2)	0.491	0.11
SPT	32.7 (6.3)	31.9 (6.2)	0.333	0.13
SASP	25.9 (5.6)	25.6 (5.6)	0.708	0.05
SPA	32.6 (6.1)	32.3 (6.0)	0.761	0.05
SSSP	21.0 (3.9)	20.8 (4.4)	0.598	0.05
Total DREEM	145.5 (22.6)	142.2 (15.8)	0.390	

*SPL *Student Perception of Learning,* SPT *Student Perception of Teachers,* SASP *Students’ academic self-perceptions,* SPA *Students’ perceptions of atmosphere*, SSSP *Students’ social self-perceptions

#### Perceptions of teaching

The mean for student perceptions of teaching was 32.71/48 for mentees while for non-mentee students the score was 31.93/48 (*P *=0.333). The strengths reported in most items were students’ positive perceptions (with mean scores more than 3) including their teachers’ constructive feedbacks, clear examples, preparation for classes, appropriate knowledge, and communication skills. Weaknesses such as teachers getting angry, humiliating the students, and their authority in the class were also addressed.

#### Academic self-perceptions

In the same breath, the overall score on this domain of the inventory was 25.95/32 for mentees while in non-mentees it was 25.71/32 (*P *=0.761). Students reported a mean score higher than 3 for all items except for item ‘‘confidence development’’ and “relevancy to future career”.

#### Perceptions of atmosphere

Mentee and non-mentee students’ social self-perception scores are very similar (mentees 32.59/48 and non-mentees 32.36/48), (*P *=0.708). Except for items “This school is well time-tabled”, “I find the experience disappointing” and “Cheating is a problem in this school”, mean scores on all other items in this subscale were higher than 3.

#### Social self-perceptions

Students’ perceptions mean scores of the social support in both student groups was approximately the same. Students with mentors had a mean of 21.04/28 and students without mentors 20.77/28 with *P *=0.598. The mean score of all items was rated more than 3 except for items “being too tired to enjoy course” and “the presence of a supportive system in time of stress”. Students as mentees expressed more perception of supportive systems in stressful situations (*P *=0.009).

## Discussion

In this study, we tried to examine the strengths and weaknesses of the new curriculum after 7 years of implementation. We also studied the perception of EE between students with mentors and non-mentees including various items affecting the educational environment for some programmatic initiatives. It is evident that mentoring programs for faculties in different aspects like students’ well-being [42], productivity [43], and feedback circle for improvements [44] positively impact the learning-teaching environment and retention of students. However, the impacts of such programs in our educational environment have not been academically studied. Our study is the first of its kind to shedlight on the issue under caption among first-year medical students’ perception of the learning environment, which compares the findings between students with and without mentors.

In line with the foregoing evidences, we have documented a high total DREEM score with more positivity than negative perception of TUMS educational environment from the perspective of first-year medical students. This concurs with the findings reported for the medical school of Lund University [45] and other studies elsewhere [46, 47]. This is probably due to the fact that in case of a favorable learning environment, students get more satisfaction with their academic placements.

Although our students’ feelings about the item “there was a good support system for students who get stressed” was acceptable, we found significant differences between mentees and non-mentees. According to some previous studies [45, 48, 49], this item seems to be a common problem in medical education and it is often attributed to the fact that medical students are suffering from stress more than other student categories, which calls for some remedial measures [50]. Many sources of stress were related to the environment of the school and the curriculum [51, 52]. It is necessary to have an innovative approach to improve the learning environment in this respect. Some studies suggested successful supporting systems for students: multiple mentors who are widely accessible [53]; mentors who with frequently remind the students about what they are expected to do [54]; mentors assigned at the right time, especially when students feel more vulnerable during transition phases [55] and the individualized support [56].

Similar to some previous studies, in our case, TUMS peer-mentors were in contact with mentees on a weekly basis from the first week of university. They were widely accessible and frequently reminded the students of what they should or should not do. TUMS peer-mentoring program implemented in a period of transition from high school to university when students had difficulties for adjustment. Our program signifies that the individualized supervision model is a crucial factor for students’ total satisfaction during their early academic training periods.

A remarkable number of students still perceived that there was too much factual content to memorize in a short time and they were too exhausted to enjoy the course. Learning the information outside the relevant context in comparison to the method in which the facts are contextualized has repeatedly been shown to be inferior. Constructivist teaching is based on being actively involved in a process of meaning-making and knowledge construction rather than passively receiving information. This type of learning fosters critical thinking and creates motivated and independent learners [57, 58]. Reform was not completely successful in reaching the goal of a more student-centered and problem-based program. Teacher-based education can be of wide spectrum. This form of education may be more or less interactive. It could be restricted just to less interactive and more lecture-based classes or on the other side of the range, it could prevent student independent action right or innovation. Our results revealed the potential effects of mentors on these stages. However, to substantiate our findings, we need further qualitative studies on different aspects of the effectiveness. Qualitative studies enable deeper understanding of students’ experiences, which cannot be easily put into numbers. Curricular changes should be handled in such a way so as to render the educational program more student-centered or problem-based.

### Strength and limitaions

One of the strengths of the present study was using a validated DREEM tool. It was also a creative study, addressing quite new concerns. The response rate in the study was also quite high. The main limitation, however, was restricting our study to the new entrants. This was done to prevent recall bias of their mentoring experience, which inevitably restricted our sample. This, in turn, might have excluded the perception of students with more negative attitudes. Other than these, the study was done in one university which might have again limited the generalizability of the results. In this study, the mentees were participated in mentoring program voluntarily which could well be a source of bias for the results interpretation.

There are problems with using Cronbach´s alpha (CA) as a quality indicator of test scores. Two strands of problems can be identified in this regard: the first strand is twofold. CA is a lower bound to the reliability, often, even a gross underestimate. Furthermore, CA cannot have a value that could be the reliability based on the usual assumptions about measurement error. Enhanced alternatives to CA exist but are little known. The second problem is that CA is insistently and erroneously taken to be a measure of the internal structure, and hence as evidence that the items “measure the same thing”. However, CA does not provide the researcher with this sort of information. Alpha values lower than 0.7 (0.6) indicate too high heterogeneity and values higher than 0.9 often indicate that the items may be too similar. Fortunately, total DREEM and its subscales had CA approximately 0.7 except for SPA with lower than 0.6.

## Conclusions

The perception of the educational environment as experienced by medical students is significantly related with having good mentors to consult with. Since having an appropriate support system was significantly different between groups with mentors and those without mentors, we suggest curriculum designers addressing the problem while reforming the programs.

Our study has proved that improvement in the educational environment can be accomplished through peer-mentoring. The study has paved the way for further research to reevaluate current study’s questions in more broadly representative samples using mixed methods and including a randomized controlled group with special emphasis on educational environment. Future studies can use more tools to confirm the role of peer-mentoring in reform processes, supplemented by focus group discussions and key informant interviews.[59].

## Supplementary Information

**Additional file 1: Table 1. **DREEM items' mean score differences between groups of students with and without a mentor.

## Data Availability

All data generated or analyzed during this review are included in this article.
