# Supplementary material for: Students’ perception of educational environment based on Dundee Ready Education Environment Measure and the role of peer mentoring: a cross-sectional study
Source: BMC Med Educ. 2022 Mar 15;22:176. doi: 10.1186/s12909-022-03219-8 (PMC8925203; doi:10.1186/s12909-022-03219-8)
Supplement: Supplementary file 1 — Additional file 1: Table 1. DREEM items' mean score differences between groups of students with and without a mentor. [file 12909_2022_3219_MOESM1_ESM.docx]

Supplementary:

Table 1: DREEM items' mean score differences between groups of students with and without a mentor

| Domain | Item | Students with mentor  Mean (SD) | Students without mentor Mean (SD) | Significance |
| --- | --- | --- | --- | --- |
| SPT | 2.The teachers are knowledgeable | 4.1 (0.7) | 4.1 (0.8) | 0.726 |
|  | 6.The teachers are patient with patients | 3.3 (0.9) | 3.3 (0.7) | 0.921 |
|  | 8.The teachers ridicule the students | 1.7 (1.1) | 1.9 (0.9) | 0.083 |
|  | 9.The teachers are authoritarian | 1.0 (0.9) | 1.0 (0.9) | 0.215 |
|  | 18. The teachers have good communications skills with patients | 3.5 (1.1) | 3.5 (1.0) | 0.539 |
|  | 29. The teachers are good at providing feedback to students | 3.2 (1.0) | 3.0 (1.0) | 0.097 |
|  | 32. The teachers provide constructive criticism here | 3.3 (1.1) | 2.9 (0.9) | 0.047 |
|  | 37. The teachers give clear examples | 3.8 (0.9) | 3.7 (1.0) | 0.265 |
|  | 39. The teachers get angry in class | 1.5 (0.9) | 1.6 (1.0) | 0.227 |
|  | 40. The teachers are well prepared for their classes | 3.8 (0.8) | 3.8 (0.9) | 0.694 |
|  | 49. The registrars irritate the course organizers | 3.2 (1.2) | 3.1 (1.1) | 0139 |
| SPL | 1. I am encouraged to participate in class | 2.8 (1.1) | 2.8 (1.3) | 0.978 |
|  | 7.The teaching is often stimulating | 2.8 (1.1) | 2.7 (1.2) | 0.436 |
|  | 13. The teaching is student-centered | 2.3 (1.1) | 2.2 (1.1) | 0.292 |
|  | 16. The teaching is sufficiently concerned to develop my competence | 3.2 (1.0) | 3.1 (1.1) | 0.432 |
|  | 20. The teaching is well focused | 3.3 (1.1) | 3.4 (1.0) | 0.844 |
|  | 21. I feel I am being well prepared for my profession | 3.1 (1.2) | 3.0 (1.1) | 0.470 |
|  | 24. The teaching time is put to good use | 3.2 (1.1) | 3.2 (1.1) | 0.997 |
|  | 25. The teaching over-emphasizes factual learning | 3.9 (0.8) | 3.9 (0.7) | 0.902 |
|  | 38. I am clear about the learning objectives of the course | 3.6 (1.1) | 3.4 (1.0) | 0.093 |
|  | 44. The teaching encourages me to be an active learner | 2.7 (1.1) | 2.7 (1.2) | 0.758 |
|  | 47. Long term learning emphasizes over short term | 2.6 (1.3) | 2.6 (1.3) | 0.936 |
|  | 48. The teaching is too teacher-centered | 0.55 (0.7) | 0.84 (0.8) | 0.037 |

SPL: Student Perception of Learning, SPT: Student Perception of Teachers

Table 1 (Continue.)

| Domain | Item | Students with mentor  Mean (SD) | Students without mentor Mean (SD) | Significance |
| --- | --- | --- | --- | --- |
| SASP | 5. Learning strategies which worked for me before continue to work for me now | 3.4 (1.1) | 3.5 (1.2) | 0.698 |
|  | 10. I am confident about my passing this year | 3.6 (1.0) | 3.8 (0.8) | 0.047 |
|  | 22. The teaching is sufficiently concerned to develop my confidence | 2.7 (1.0) | 2.7 (1.1) | 0.981 |
|  | 26. Last year’s work has been a good preparation for this year’s work | 3.1 (1.0) | 3.1 (1.1) | 0.831 |
|  | 27. I am able to memorize all I need | 3.2 (1.2) | 3.2 (1.1) | 0.766 |
|  | 31. I have learned a lot about empathy in my profession | 3.2 (1.1) | 3.3 (1.1) | 0.537 |
|  | 41. My problem-solving skills are being well developed here | 3.3 (1.1) | 3.1 (1.1) | 0.131 |
|  | 45. Much of what I have to learn seems relevant to a career in healthcare | 3.0 (1.2) | 3.0 (1.3) | 0.934 |
| SPA | 11. The atmosphere is relaxed during the ward teaching | 3.2 (1.2) | 3.0 (1.2) | 0.128 |
|  | 12. This school is well time-tabled | 2.6 (1.3) | 2.2 (1.1) | 0.037 |
|  | 17. Cheating is a problem in this school | 1.5 (1.0) | 1.6 (0.9) | 0.073 |
|  | 23. The atmosphere is relaxed during lectures | 3.1 (1.1) | 3.1 (1.2) | 0.879 |
|  | 30. There are opportunities for me to develop interpersonal skills | 3.4 (1.2) | 3.2 (1.0) | 0.131 |
|  | 33. I feel comfortable in class socially | 3.1 (1.2) | 3.1 (1.1) | 0.626 |
|  | 34. The atmosphere is relaxed during seminars/tutorials | 3.6 (1.0) | 3.6 (0.9) | 0.469 |
|  | 35. I find the experience disappointing | 1.2 (1.0) | 1.5 (1.0) | 0.015 |
|  | 36. I am able to concentrate well | 3.2 (1.1) | 3.1 (1.0) | 0.401 |
|  | 42. The enjoyment outweighs the stress of the course | 3.1 (1.2) | 3.0 (1.2) | 0.587 |
|  | 43. The atmosphere motivates me as a learner | 2.9 (1.2) | 3.0 (1.0) | 0.430 |
|  | 50. The students irritate the teachers | 1.4 (1.0) | 1.5 (1.1) | 0.204 |
| SSSP | 3. There is a good support system for students who get stressed | 2.9 (1.2) | 2.6 (1.1) | **0.009*** |
|  | 4. I am too tired to enjoy the course | 1.1 (1.0) | 1.1 (1.0) | 0.700 |
|  | 14. I am rarely bored on this course | 2.7 (1.0) | 2.6 (1.0) | 0.696 |
|  | 15. I have good friends in this school | 4.1 (1.0) | 4.1 (0.9) | 0.939 |
|  | 19. My social life is good | 3.7 (1.0) | 3.8 (0.9) | 0.465 |
|  | 28. I seldom feel lonely | 3.3 (1.2) | 3.1 (1.3 ) | 0.227 |
|  | 46. My accommodation is pleasant | 3.2 (1.1) | 3.2 (1.3) | 0.493 |

SASP: Students’ academic self-perceptions, SPA: Students’ perceptions of atmosphere, SSSP: Students’ social self-perceptions *Items with P value <0.001 was selected as statistically significance.
